# Altered gut microbial functional landscape in children with pediatric inflammatory multisystem syndrome following SARS-CoV-2 infection: an exploratory metagenomic study

**DOI:** 10.3389/fped.2026.1881538

**Published:** 2026-09-21

**Authors:** J. Agrimbau Vázquez, C. Boggio Marzet, R. Peralta, R. Taussig, P. Lopez, D. Viale, C. Alonso, T. Curtti, M. L. Perez Gagni, M. L. Cassará, L. Urrutia, J. P. Bustamante

**Affiliations:** 1 Hospital de Pediatría “Prof. Dr. J. P. Garrahan”, Buenos Aires, Argentina; 2 Facultad de Medicina, Universidad de Buenos Aires Buenos Aires, Argentina; 3 Hospital General de Agudos “Dr. I. Pirovano”, Buenos Aires, Argentina; 4 Facultad de Ingeniería, Universidad Austral, Laboratorio de Investigación, Desarrollo y Transferencia de la Universidad Austral (LIDTUA-CIC), Pilar, Argentina; 5 Facultad de Ingeniería, Universidad Nacional de Entre Ríos, Oro Verde, Argentina; 6 Facultad de Ciencias Bioquímicas y Farmacéuticas, Universidad Nacional de Rosario (UNR) Rosario, Argentina; 7 Fundación Pablo Cassará, Buenos Aires, Argentina; 8 Centro Interdisciplinario de Investigaciones en Ciencias de la Salud y del Comportamiento (CIICSAC, CONICET-UAP), Facultad de Ciencias de la Salud, Universidad Adventista del Plata, Entre Ríos, Argentina

**Keywords:** COVID-19, gut microbiota, gut–lung axis, metagenomics, MIS-C, pediatric inflammatory multisystem syndrome, PIMS, SARS-CoV-2

## Abstract

**Background:**

Pediatric Inflammatory Multisystem Syndrome (PIMS), also known as MIS-C (Multisystem inflammatory syndrome in children), is a severe post-infectious inflammatory condition associated with SARS-CoV-2 in children. While coronavirus disease 2019 (COVID-19), caused by SARS-CoV-2, primarily affects the respiratory system, growing evidence highlights gastrointestinal involvement and the relevance of the gut–lung axis in systemic inflammation. However, the taxonomic and, particularly, the functional landscape of the gut microbiome in children with PIMS remains insufficiently characterized.

**Methods:**

This exploratory study analyzed fecal samples from pediatric patients diagnosed with PIMS and age-matched clinically healthy controls using shotgun metagenomic sequencing. Taxonomic profiling was performed with MetaPhlAn4, and functional and metabolic pathway analyses were conducted using HUMAnN3. Alpha and beta diversity metrics were assessed, and differential abundance analyses were applied to identify microbial taxa and putative functional pathways associated with PIMS.

**Results:**

12 pediatric patients diagnosed with PIMS and 11 age-matched clinically healthy controls were included. Alpha diversity indices did not differ significantly between groups, although consistently lower mean values were observed in children with PIMS. In contrast, beta diversity analysis demonstrated a significant separation in microbial community composition between patients with PIMS and controls (PERMANOVA, *p* = 0.01). Children with PIMS exhibited increased relative abundance of *Prevotella copri* clade C, *Duodenibacillus massiliensis*, *Phascolarctobacterium succinatutens*, and *Enterocloster bolteae*, alongside a relative reduction of several commensal taxa. Putative functional profiling revealed significant differences in enzyme-coding genes and metabolic pathways, including increased metagenomic abundance of aconitate hydratase and other functions potentially relevant to inflammatory and immunomodulatory processes in the PIMS group.

**Conclusion:**

These findings suggest an association between gut microbiota unbalance, potential microbial functional alterations, and PIMS, supporting the need for further investigation of the gut microbiome in post-COVID-19 systemic inflammation in pediatric populations. Given the exploratory nature of this study, these observations require validation in larger, longitudinal cohorts before microbial biomarkers or therapeutic implications can be established.

## 1 Introduction

Since the beginning of the coronavirus disease 2019 (COVID-19) pandemic, some children have had severe sequelae after infection, defined as Pediatric Inflammatory Multisystem Syndrome (PIMS) associated with SARS-CoV-2 infection or Childhood Multisystem Inflammatory Syndrome (MIS-C). This syndrome is a novel condition first reported in April 2020, characterized by the presence of fever, evidence of inflammation in laboratory tests, multiorgan involvement, acute malaise, and infection or exposure to SARS-CoV-2. Other clinical features include acute myocardial dysfunction, respiratory failure, Kawasaki disease-like manifestations, and toxic shock syndrome-like symptoms (1–6). These multisystem inflammatory diseases are emerging disorders that result from an unusual response to SARS-CoV-2 infection, which is mediated by the host's innate and adaptive immune systems (7).

PIMS is a new entity with very low prevalence, but it carries a significant risk of complications, complex hospitalizations, and unknown long-term sequelae. The current prognosis for PIMS is good, with reports of low mortality (approximately 1%–2%), although late diagnosis could worsen outcomes (8). More than half of affected children require admission to a pediatric intensive care unit, reflecting the severity of the syndrome despite its rarity, which are sometimes limited in general hospitals (8, 9).

First-line treatment of PIMS with either glucocorticoids alone or intravenous immunoglobulin (IVIG) plus glucocorticoids showed similar effectiveness in preventing severe outcomes (mechanical ventilation, inotropic support, or death) compared to IVIG alone. The use of glucocorticoids alone was an acceptable, effective and initial treatment (10).

An essential interaction between the mucosal tissues of the gastrointestinal and respiratory systems has been observed, as illustrated by intestinal complications during respiratory diseases and vice versa. Despite the lack of a detailed understanding of its mechanism, the concept of the gut-lung axis and its importance for both well-being and disease is critical for understanding the causes and treatment of disease (11). Following this idea, despite the fact that COVID-19 is primarily a respiratory disease, there is increasing evidence to suggest that the gastrointestinal tract is involved in this pathology. A meta-analysis reported that up to 20% of COVID-19 patients had gastrointestinal symptoms (12). In addition, SARS-CoV-2 binds to angiotensin-converting enzyme 2 (ACE2) receptors to invade human cells, and these receptors are largely expressed in the intestinal epithelium (13–15). In contrast, viral RNA and activated viruses were detected in fecal samples, suggesting that the digestive tract could be a site of viral replication and activity (16). Taken together, these observations provide the rationale for investigating the gut microbiota and its functional potential in children with PIMS following SARS-CoV-2 infection.

Current diagnosis and clinical assessment of PIMS rely on inflammatory and cardiac biomarkers, including erythrocyte sedimentation rate (ERP), C-reactive protein (CRP), increased levels of neutrophilia, ferritin, troponin, fibrinogen, and D-dimer, elevated values of type B natriuretic (BNP), liver function tests, and conventional blood markers (9, 17–21). However, these biomarkers primarily reflect systemic inflammation and tissue injury rather than biological mechanisms potentially involved in disease development. Consequently, additional biomarkers capable of providing mechanistic insights, including microbiome-derived features, may complement current clinical evaluation.

In the present study, we intentionally use the term gut microbiota unbalance rather than dysbiosis. Although dysbiosis is widely used in microbiome research, there is currently no universally accepted definition or standardized diagnostic criteria for this concept, and its interpretation remains context-dependent (22). Therefore, we adopted the more descriptive expression gut microbiota unbalance to refer to compositional and functional alterations observed in the microbial community without implying the existence of a universally defined pathological microbiome state.

The connection between SARS-CoV-2 and the digestive system, particularly the interaction of the virus with the gut microbiota, was studied worldwide. This raises the need and interest to examine the role and behavior of the microbiota in the evolution of PIMS. Most available studies have relied on 16S rRNA gene sequencing or targeted molecular approaches, which are useful for describing community composition but provide limited direct information on the microbial gene repertoire. Consequently, it remains unclear whether PIMS is associated with distinct microbial enzyme-coding genes, metabolic pathways, or other functional features potentially relevant to systemic inflammation and immune dysregulation.

Although changes in microbial composition have been reported in several inflammatory and infectious diseases, taxonomic information alone provides only a partial understanding of microbiome-host interactions. Despite the fact that previous studies have identified gut microbial compositional alterations in children with SARS-CoV-2 infection and PIMS, the functional potential of these microbial communities remains insufficiently characterized (23). Many biological effects of the gut microbiota are mediated through its metabolic activities, including the production of bioactive metabolites, modulation of immune signaling pathways, nutrient metabolism, and regulation of epithelial barrier function. Therefore, characterizing the functional potential encoded by the gut microbiome may provide mechanistic insights beyond taxonomic shifts alone. Functional information has also been explored through metaproteomics in pediatric COVID-19, although not specifically in children with PIMS (24). In this sense, shotgun metagenomic sequencing enables simultaneous characterization of microbial community composition and potential functional gene content, allowing the identification of metabolic pathways and enzyme-coding genes potentially associated with disease-associated inflammatory processes. Given that PIMS is characterized by an exaggerated systemic immune response rather than by direct viral damage (25), exploring both the taxonomic and functional landscape of the gut microbiome may contribute to identifying microbial features associated with this condition and generate hypotheses regarding microbiome-host interactions involved in its pathophysiology. Unlike amplicon-based approaches that are primarily restricted to taxonomic characterization, shotgun metagenomics enables the simultaneous investigation of microbial composition and predicted functional capacity. This integrated approach was therefore selected to better explore the inferred functional potential of the gut microbiome to the immunometabolic disturbances that characterize PIMS and represents, to our knowledge, one of the first exploratory shotgun metagenomic studies addressing both taxonomic and putative functional alterations in pediatric PIMS from Latin America.

## 2 Materials and methods

### 2.1 Experimental design and patient enrollment

This exploratory case-control study included all consecutive pediatric patients diagnosed with PIMS at Hospital de Pediatría “Prof. Dr. J. P. Garrahan” who fulfilled the predefined eligibility criteria between June 1, 2020, and June 1, 2022. Hospital Garrahan is the largest public tertiary pediatric referral hospital in Argentina and one of the national referral centers for pediatric COVID-19. Because PIMS is a rare complication of SARS-CoV-2 infection in children, the study cohort was determined by the number of eligible cases available during the study period rather than by a prespecified sample size. Among 62 PIMS cases evaluated at the institution during this period, 12 fulfilled the study eligibility criteria and were included in the present analysis.

Eligible participants were children aged 2–16 years who met the WHO and CDC diagnostic criteria for PIMS. Exclusion criteria were: (1) identification of an alternative diagnosis following the initial diagnostic evaluation; (2) use of antibiotics within 7 days prior to admission and/or oral corticosteroids within the month prior to admission; (3) primary or secondary immunodeficiency (including HIV), juvenile idiopathic arthritis, severe/morbid obesity, chronic lung, kidney, or liver disease, previous Kawasaki disease, or neoplastic disease; and (4) inability to comply with the study protocol.

Among the 12 patients with PIMS, six (50%) were female and six (50%) were male, with a mean age of 7.9 years (range, 1.6–13.8 years). Nine patients (75%) had pre-existing comorbidities: non-severe obesity (*n* = 5), genetic syndrome (*n* = 1), previous neurological disease (*n* = 1), branchio-oto-renal syndrome (*n* = 1), and type 1 diabetes (*n* = 1). Previous SARS-CoV-2 infection was documented by PCR and/or antigen testing in 33.4% of patients, while 16.6% met the clinical criterion with positive SARS-CoV-2 serology.

The control group consisted of 11 age-matched clinically healthy children recruited during the same study period. All were from a middle socioeconomic background and followed a Western dietary pattern. None had clinical evidence of SARS-CoV-2 infection at enrollment, inflammatory gastrointestinal disease, chronic systemic disease, or antimicrobial exposure during the preceding 7 days, and none had received corticosteroid treatment during the 30 days prior to sample collection. Control participants underwent fecal sample collection exclusively for microbiome analysis and were not subjected to the additional clinical investigations performed in the PIMS cohort.

The group of patients underwent multiple clinical studies at the time of admission and subsequent hospitalization (including routine check-ups, blood pressure, body temperature, abdominal ultrasound, blood and urine tests, and COVID-19 test), along with fecal extraction for microbiota analysis. The healthy group only had fecal matter extracted for microbiota analysis.

All participants and/or their legal guardians read and signed the written informed consent prior to enrollment, in accordance with the institutional guidelines of Hospital de Pediatría “Prof. Dr. J. P. Garrahan”. The informed consent form and study protocol were approved by the Hospital de Pediatría “Prof. Dr. J. P. Garrahan” Ethics Committee.

### 2.2 Microbiota analysis

The ZymoBIOMICS™ DNA Miniprep kit was used to extract genetic material from fecal samples. Once the extraction was complete, the concentration was measured using a Qubit® 4 fluorometer and a dsDNA high-sensitivity kit. An aliquot of the extract was analyzed using an electrophoretic gel to confirm that the DNA had not degraded during the process.

From the extracted DNA, libraries were prepared according to the Illumina® DNA Prep Reference Guide using the following kits: Illumina® DNA Prep PCR+Buffers, Illumina® DNA Prep Beads+Buffers, IDT® for Illumina® DNA/RNA UD Index Set A, and Illumina® DNA Prep Tagmentation (M) Beads. The concentration of the libraries was also measured using a Qubit® 4 fluorometer and a dsDNA high-sensitivity kit. Quality was measured using an Agilent Bioanalyzer 2100® with the High Sensitivity DNA Kit.

The sequencing of the genetic material of each sample was carried out using a NextSeqTM 500 sequencer according to the protocol established by Illumina®.

Once the sequences were obtained, quality control was performed using Trimmomatic (26). Kneaddata (https://bitbucket.org/biobakery/kneaddata/wiki/Home) was used to remove human DNA sequences in samples with the human genome grch38 as a reference (https://www.ncbi.nlm.nih.gov/genome/guide/human/).

MetaPhlAn4 (27) was used to obtain the taxonomic profile, which aligns metagenome reads with a predefined database of marker genes for taxonomic classification.

Humann3 (28) was used to obtain the functional profile, which also allows exploring intra- and inter-sample contributory diversity (contributions of species to a specific function) using the KEGG (29), COG (30), eggNOG (31), Metacyc (32), and Pfam (33) databases and the four levels of the Enzyme Commission number (EC number).

### 2.3 Sequence quality

Reads were quality- and length-trimmed with Trimmomatic (SLIDINGWINDOW:4:20, minimum quality score 20 over a 4-base window, minimum retained length 58 bp), and human-derived reads were removed with Kneaddata/Bowtie2 (–very-sensitive mode) against the GRCh37 reference genome. Across the 23 samples processed in the final analytic set, a mean of 13,069,574 raw paired-end reads per sample (range 5,103,355–20,172,797) was generated; after quality/length trimming, a mean of 9,808,033 paired reads per sample were retained (74.9% of raw reads); after removal of human-derived reads (median 1.0% of trimmed reads), a mean of 8,955,022 quality-filtered, non-human paired reads per sample (range 2,170,521–15,079,058) was retained for downstream taxonomic and functional profiling.

### 2.4 Statistical analysis

The analysis of alpha and beta diversity (Bray-Curtis distance matrix) was performed with MicrobiotaProcess (34), the Mann–Whitney test was used to evaluate the differences between the alpha diversity metrics: Observed, chao1, Ace, Shannon, Simpson, Pielou and Fisher. To evaluate the differences between the groups based on the results of beta diversity, the PERMANOVA test (35) implemented in the adonis() function of the vegan package of R (https://github.com/vegandevs/vegan) was used. To identify specific microbial taxa and metabolic pathways that consistently differ between groups, we employed LEfSe (Linear discriminant analysis Effect Size) (36). LEfSe is specifically designed for high-dimensional metagenomic comparisons. It first uses the non-parametric Kruskal–Wallis test to detect features with significant differential abundance. Subsequently, it performs pairwise Wilcoxon tests among subclasses to ensure the detected differences are biologically consistent. Finally, it estimates the effect size of each significant feature using Linear Discriminant Analysis (LDA), providing a ranking of the most relevant biomarkers that explain the differences between the PIMS and control groups.

LDA scores were computed using a threshold of |LDA| ≥ 2.0, the default value proposed in the original LEfSe framework. No additional correction for multiple comparisons was applied beyond the internal robustness checks built into the LEfSe algorithm itself (non-parametric Kruskal–Wallis test followed by pairwise Wilcoxon consistency testing across subclasses prior to LDA), consistent with the exploratory, hypothesis-generating design of this pilot study. Given the limited sample size (12 children with PIMS vs. 11 controls) relative to the high dimensionality of metagenomic and functional feature space, a strict multiple-testing correction (e.g., Benjamini-Hochberg FDR) would be expected to substantially reduce statistical power and was considered overly conservative for an initial characterization of candidate taxa and pathways. Findings should therefore be interpreted as preliminary and hypothesis-generating, warranting confirmation in larger, independent cohorts.

## 3 Results

### 3.1 Clinical evaluation and laboratory findings of children with PIMS

The main demographic, epidemiological, clinical, laboratory, imaging, and treatment characteristics of the 12 children with PIMS are summarized in Table 1. Overall, the cohort showed the multisystem clinical presentation characteristic of PIMS, with prominent gastrointestinal manifestations and systemic inflammation, while severe respiratory or cardiovascular support was not required. Clinical and cardiac follow-up was conducted for up to two months, with no persistent clinical abnormalities or laboratory evidence of ongoing cardiac or inflammatory involvement.

**Table 1:** Demographic, epidemiological, clinical, laboratory, imaging, and treatment characteristics of the 12 children with PIMS.

Variable	Result
Patients included	12
Mean age	7.9 years (range 1.6–13.8)
Female sex	6 (50%)
Male sex	6 (50%)
Pre-existing comorbidities	7 (58.3%)
Obesity	5
Genetic syndrome	1
Previous neurological disease	1
Branchio-oto-renal (BOR) syndrome	1
Type 1 diabetes mellitus	1
Epidemiological link as enrollment criterion	6 (50%)
Previous positive PCR/antigen test as enrollment criterion	4 (33.3%)
Positive serology as enrollment criterion	2 (16.6%)
Fever	12 (100%)
Mean time from fever onset to diagnosis	4.6 days
Abdominal pain	9 (75%)
Vomiting and diarrhea	6 (50%)
Diarrhea alone	2 (16.6%)
Conjunctivitis	6 (50%)
Skin rash	5 (41.6%)
Oral mucosal changes	3 (25%)
Extremity changes	2 (16.6%)
Upper respiratory tract symptoms	2
Seizures and irritability	1
Kawasaki phenotype	1
Myocarditis	2 (16.6%)
Coronary artery involvement	0
Anemia	7 (58.3%)
Lymphopenia	6 (50%)
Thrombocytopenia	5 (41.6%)
Elevated CRP	12 (100%); mean 181.9 (range 40–399)
Positive SARS-CoV-2 PCR at some point during hospitalization	6 (50%)
Reactive SARS-CoV-2 IgG	12 (100%)
Non-reactive SARS-CoV-2 IgM	12 (100%)
Blood cultures performed	10
Positive blood cultures	0
Positive stool cultures/virological studies	0
Abdominal ultrasound performed	8
Abnormal abdominal ultrasound	4 (33.3%)
Shock at admission	2: 1 hypovolemic and 1 cardiogenic
Fluid resuscitation	3
Oxygen therapy for 24 h	2
Mechanical ventilation	0
ICU admission	0
IVIG (intravenous immunoglobulin) treatment	12 (100%)
Favorable response to IVIG	Mean time 1.4 days (range 0–7)
Intravenous corticosteroids	2
Broad-spectrum antibiotic therapy	8; mean duration 3 days
Antibiotics used	Mainly ceftriaxone in combination with clindamycin and/or vancomycin

### 3.2 Microbial diversity between patients and control groups

Several alpha diversity metrics were computed (Observed, chao1, Ace, Shannon, Simpson, Pielou, and Fisher). Looking at them, no statistically significant differences were observed. However, the mean values of the metrics for the children with PIMS were lower than those of the control group (Figure 1). In contrast, statistically significant differences were observed between the groups by analyzing their taxonomic composition (beta diversity) (PERMANOVA: statistic *R* = 1.82, *p*-value = 0.01).

**Figure 1:**
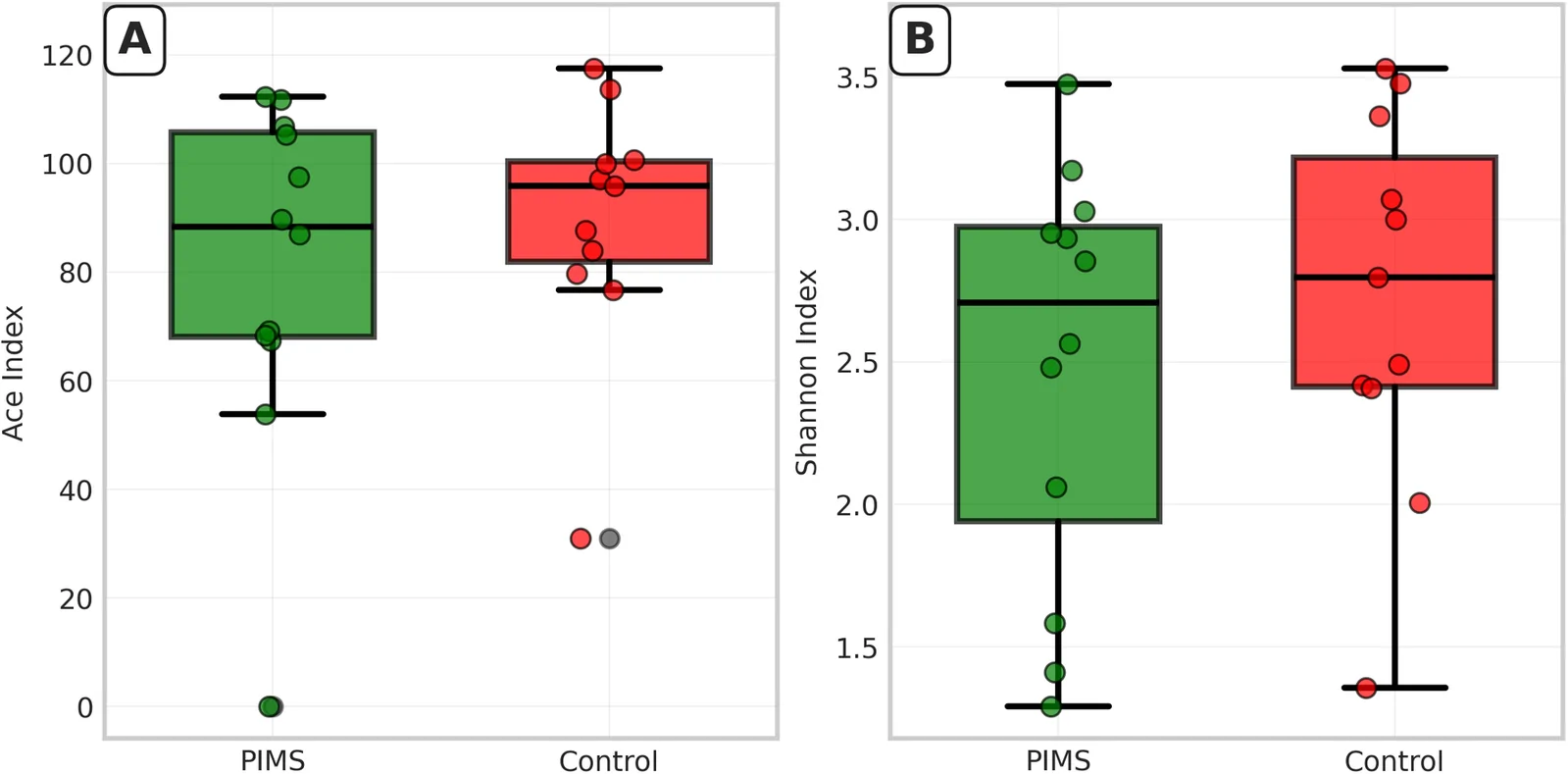
Box plots for the alpha diversity metrics of **(A)** Ace and **(B)** Shannon for the children with PIMS (green) and control (red) groups. No statistically significant differences were observed. Mean values of the metrics for the children with PIMS were lower than those of the control group.

### 3.3 Relevant differences in particular observed microbes between both groups

In both groups, approximately 50% of the microbial diversity was accounted for by 10 species commonly found in the gut (Figure 2).

**Figure 2:**
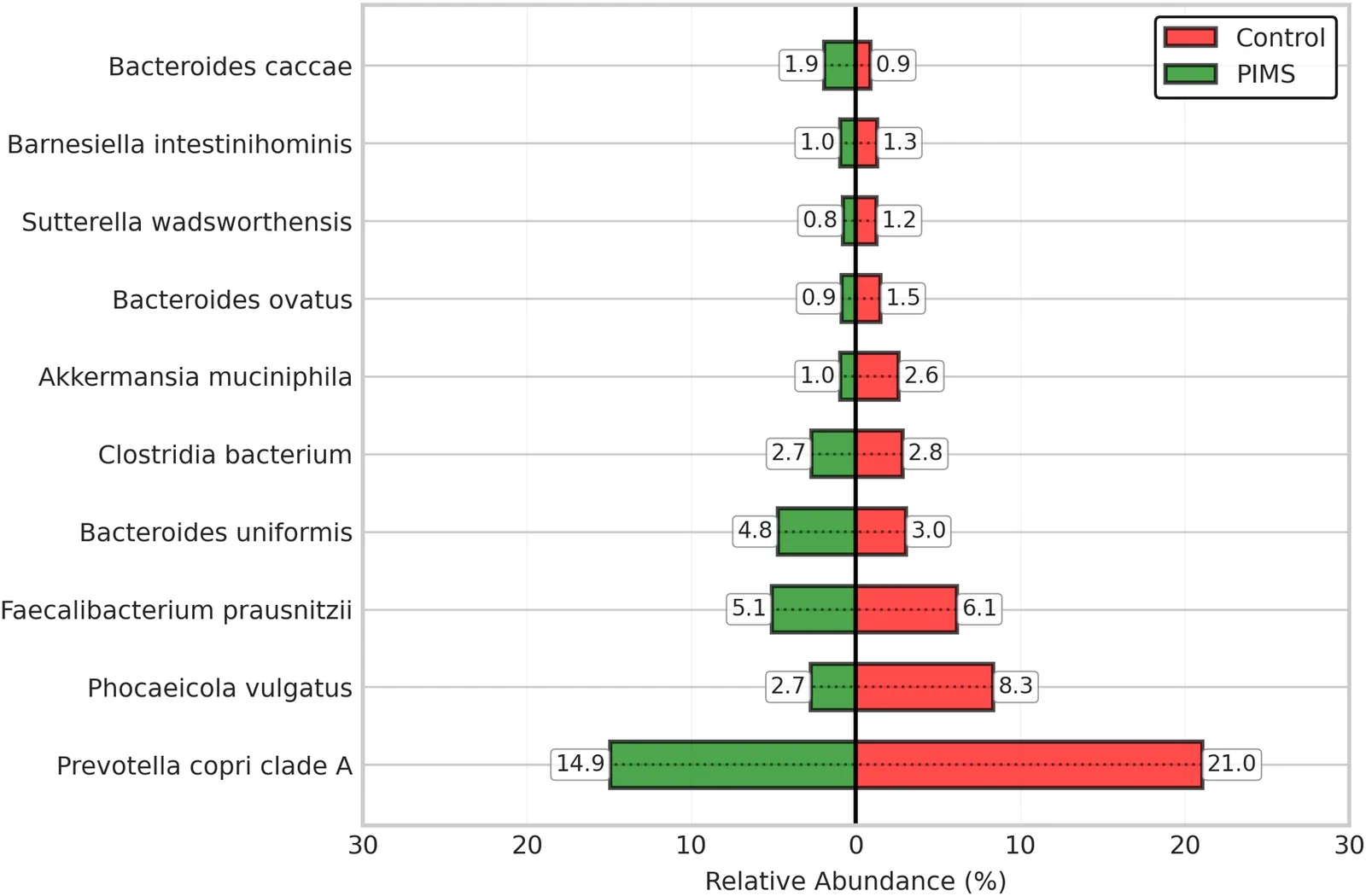
The 10 most abundant bacteria on average in both groups, children with PIMS (green) and control (red).

For the control group, the bacterial species that showed higher relative abundances with statistically significant differences in relation to the children with PIMS were *Phocaeicola vulgatus, Alistipes putredinis, Candidatus Cibiobacter qucibialis, Lachnospira eligens*, species of the genus Oscilliacter, and species of the family Ruminococcaceae (Figure 3). All are common microbes of the human gut.

**Figure 3:**
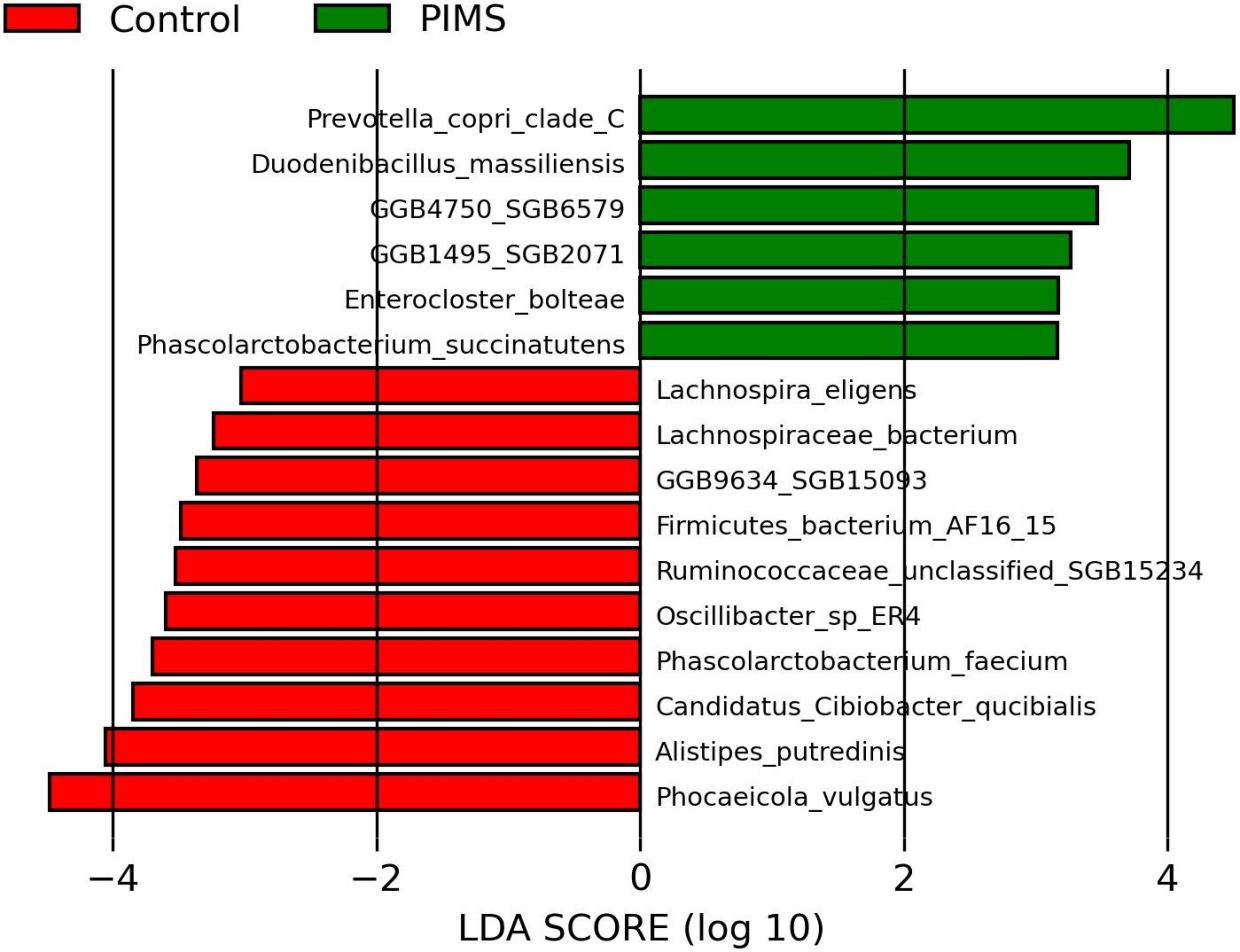
Microbial species with statistically significant differences between both groups, children with PIMS (green) and control (red).

In the PIMS group, *Prevotella copri* clade C, *Duodenibacillus massiliensis*, *Phascolarctobacterium succinatutens*, and *Enterocloster bolteae* were all significantly increased in relative abundance compared to controls (Figure 3).

### 3.4 Key functional and metabolic differences between the control group and children with PIMS

Based on the analysis of enzymes at EC level 4, we observed that, within the PIMS group, several proteins exhibited statistically significant differences, EC.4.2.1.3 (aconitate hydratase) (Figure 4), EC.1.1.1.18 (inositol 2-dehydrogenase), and EC.4.1.1.49 [phosphoenolpyruvate carboxykinase (ATP)] (Figure 5). On the other hand, for the control group, the enzymes with relevant differences in terms of their abundance in the samples were EC.2.7.1.50 (hydroxyethylthiazole kinase), EC.2.7.7.85 (diadenylate cyclase), EC.2.1.1.33 (tRNA (guanine(46)-N(7))-methyltransferase), EC.3.1.3.48 (protein-tyrosine-phosphatase) (Figure 6), EC.3.2.1.89 (arabinogalactan endo-beta-1,4-galactanase) and EC.3.1.11.6 (exodeoxyribonuclease VII) (Figure 5).

**Figure 4:**
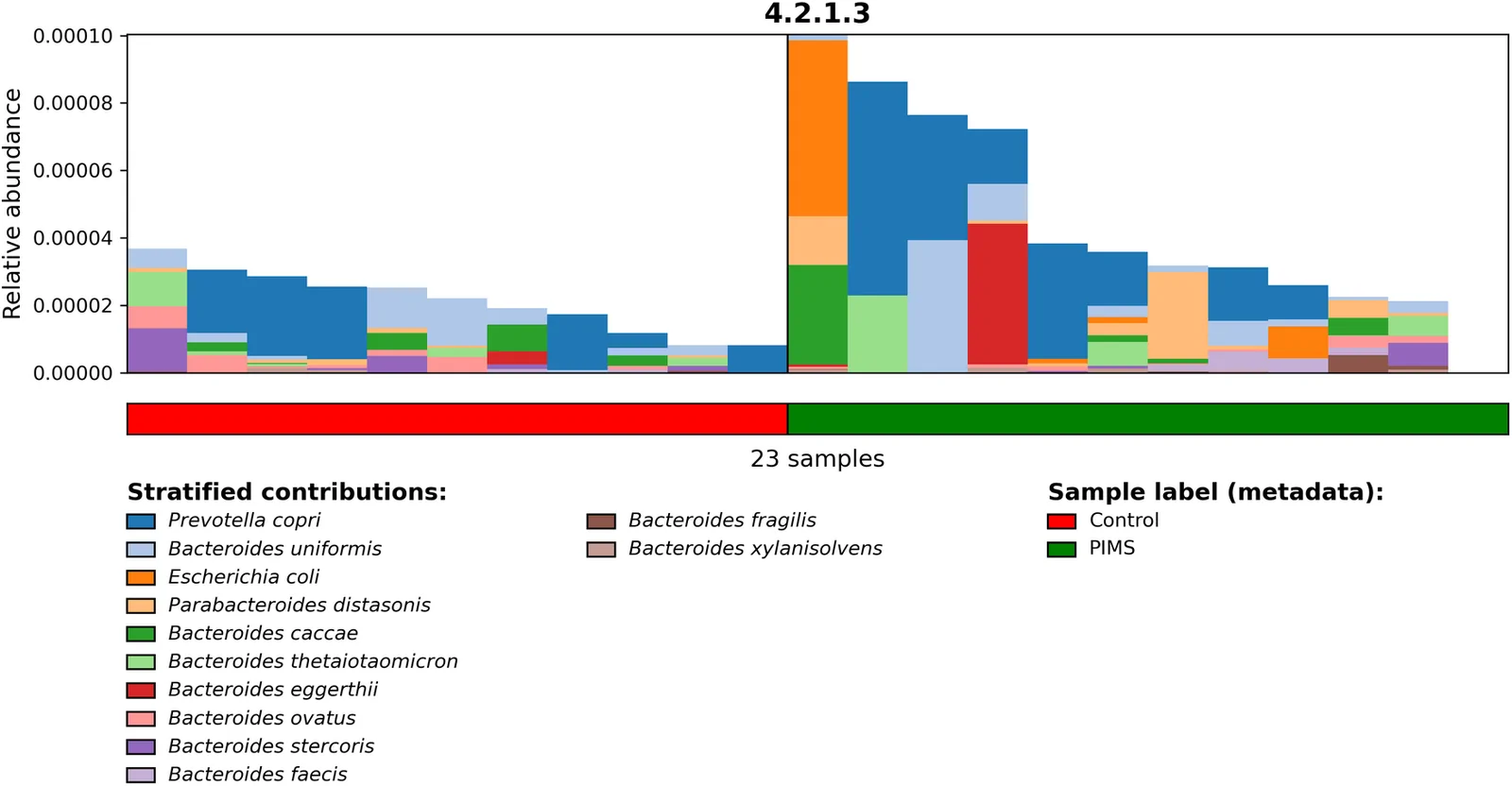
Relative abundance of identified coding genes for EC.4.2.1.3 (aconitate hydratase), stratified by group, sample and contributing microbes, with a higher prevalence in the children with PIMS group (green).

**Figure 5:**
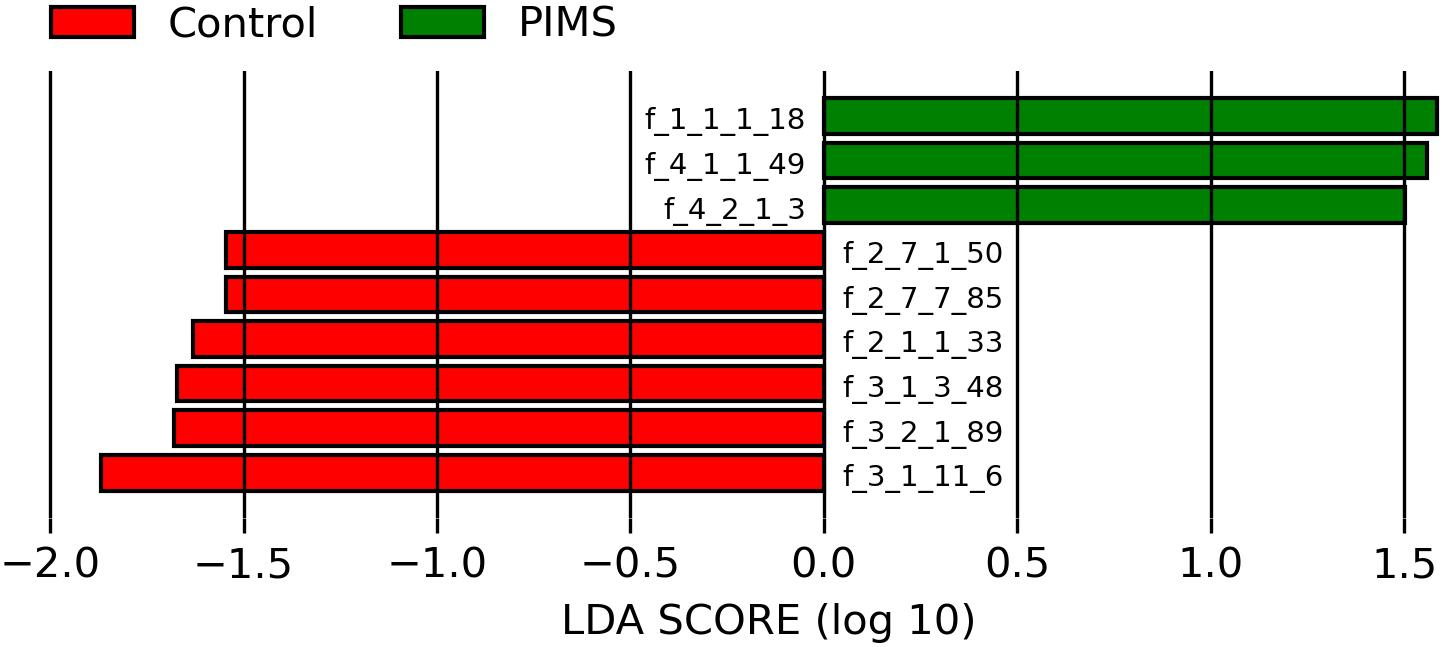
Enzymes with statistically significant differences between children with PIMS (green) and control (red) groups based on classification by EC number.

**Figure 6:**
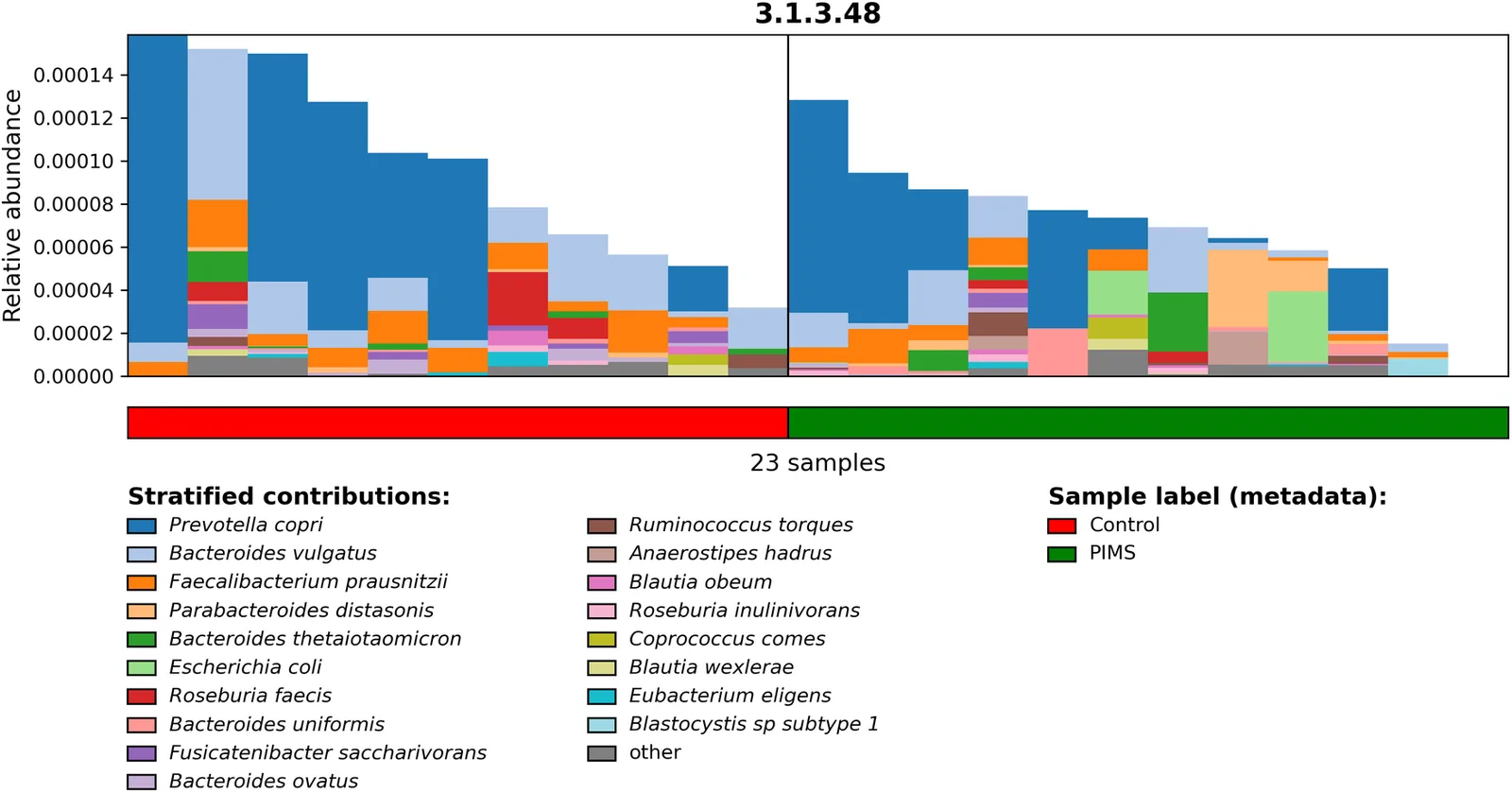
Relative abundance of identified coding genes for EC.3.1.3.48 (protein-tyrosine-phosphatase), stratified by group, sample and contributing microbes, with a higher prevalence in the control group (red).

From the Gene Orthology database (COG database), significant differences were obtained between groups for the cog1977 entry (molybdopterin synthase sulfur carrier subunit MoaD), with greater presence in the patients group, and cog3344 (retron-type reverse transcriptase) with a higher level in the control group. In contrast, for the Gene Ontology database, the entries GO:0006270 (DNA replication initiation), GO:0009279 (cell outer membrane), GO:0015562 (efflux transmembrane transporter activity), and GO:0008134 (transcription factor binding) were significantly more present in the group of patients with PIMS, and GO:0015986 (proton motive force-driven ATP synthesis) was present in the healthy or control group (Figure 7).

**Figure 7:**
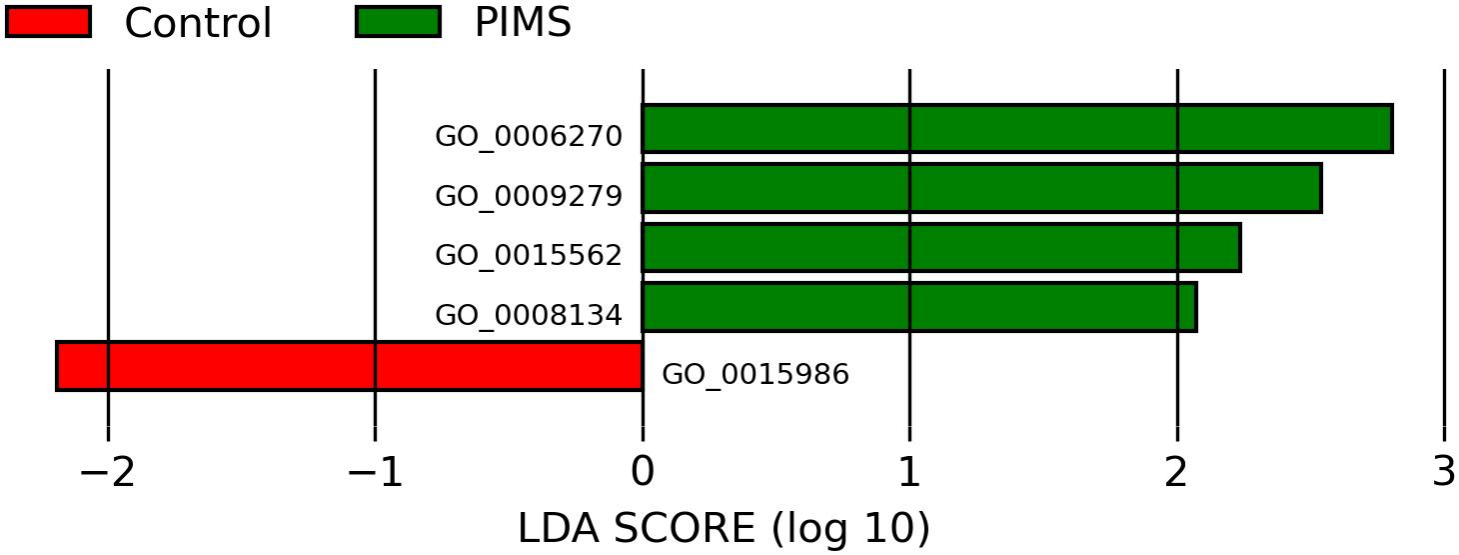
Ontology of genes with statistically significant characteristics (entries) between children with PIMS (green) and control (red) groups.

In addition, looking at mapping genes with the KEGG database showed that the K00567 entry (methylated-DNA-[protein]-cysteine S-methyltransferase) has a higher abundance in the group of patients, with K03711 (Fur family transcriptional regulator, ferric uptake regulator), K02428 (fatty acyl-AMP ligase FadD32) and K06919 (putative DNA primase/helicase) showing a greater presence in the control group.

The putative metabolic pathways obtained with humann3 from the MetaCyc database showed that the PWY-6703 (preQ0 biosynthesis) and PWY-7204 (pyridoxal 5'-phosphate biosynthesis II) pathways were more abundant in the patient group. In contrast, the PWY-7356 (thiamine diphosphate salvage IV) and PWY-6807 [xyloglucan degradation II (exoglucanase)] pathways showed higher levels in the control group (Figure 8).

**Figure 8:**
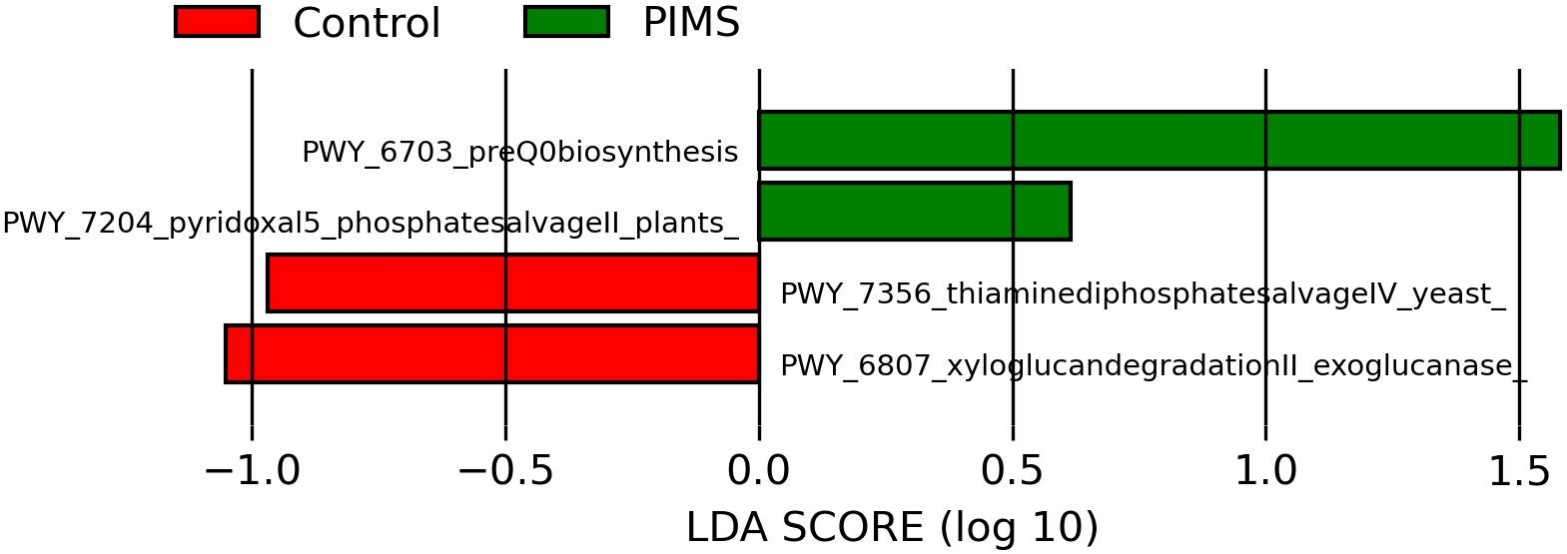
Putative metabolic pathways with significant differences between children with PIMS (green) and control (red) groups.

## 4 Discussion

To our knowledge, this study represents one of the first exploratory shotgun metagenomic analyses integrating both taxonomic and functional profiling of the gut microbiome in children with PIMS, providing complementary information beyond microbial composition alone. It is important to acknowledge that the PIMS cohort included in this study was relatively small, comprising 12 patients and 11 clinically healthy controls. However, considering that these were pediatric patients with a recent history of COVID-19 and undergoing clinical interventions, the assembly of this cohort for an exploratory human microbiome study remains highly valuable.

The relatively small cohort also represents the principal methodological limitation of this study. This sample size was primarily determined by the epidemiology of PIMS and the availability of eligible patients rather than by an *a priori* power calculation. Hospital de Pediatría “Prof. Dr. J. P. Garrahan”, where this study was conducted, is the largest public tertiary pediatric referral hospital in Argentina and served as one of the national referral centers for pediatric COVID-19 and PIMS during the pandemic. Between 2020 and 2023, a total of approximately 60 pediatric patients with COVID-19 meeting the study recruitment criteria were evaluated at our institution, of whom 12 fulfilled the diagnostic criteria for PIMS and were included in this study. Therefore, the analyzed cohort represents essentially the complete series of eligible PIMS cases recruited at our institution during the study period. With 12 children with PIMS and 11 clinically healthy controls, statistical power is inherently constrained in this exploratory pilot study by the high dimensionality of shotgun metagenomic taxonomic and functional datasets. Accordingly, differential abundance analyses performed with LEfSe were intended to identify candidate microbial taxa and functional features in an exploratory, hypothesis-generating context. Although LEfSe incorporates sequential non-parametric statistical testing and biological consistency assessment before estimating effect sizes, no additional correction for multiple comparisons (e.g., Benjamini-Hochberg false discovery rate) was applied, as such procedures would be expected to substantially reduce statistical power in a pilot study of this size. Consequently, the taxa, enzyme-coding genes, and metabolic pathways identified here should be interpreted as preliminary candidate biomarkers requiring confirmation in larger, adequately powered and independent cohorts.

Regarding the clinical characteristics associated with hospitalization in PIMS, it is noteworthy that none of the patients in our study required transfer to the intensive care unit for life support or mechanical ventilation. Other studies are focused only in patients with PIMS treated in ICUs or (37) have a population close to 50% that received this particular care (38–40). Regarding gastrointestinal system-related characteristics, in all the analyzed studies, more than half of the patients with PIMS presented abdominal pain, vomiting, and/or diarrhea (37–40), which is a differential factor from Kawasaki disease (41). Other symptomatological scenarios in the studied pathology are conjunctivitis and skin rashes, both of which are present in approximately half of the patients analyzed in various studies (37, 39, 40). Regarding the clinical laboratory findings, it is important to highlight the elevated levels of CRP, a key molecular marker of systemic inflammation, observed in all analyzed patients. Elevated levels of BNP and pro-BNP were also detected, both recognized biomarkers of cardiac dysfunction. Nevertheless, reported cardiac involvement did not exceed 50% of cases in any of the studies analyzed (37–40).

In this context of systemic inflammation and variable cardiac involvement, the gut microbiota profile revealed notable compositional differences between children with PIMS and controls, which should be interpreted in the context of previous pediatric studies. We observed no statistically significant differences in alpha diversity, although mean values were consistently lower in the PIMS group, whereas beta-diversity analysis demonstrated significant separation between children with PIMS and healthy controls. Previous studies have also reported altered community structure in children with PIMS, although the direction and magnitude of alpha-diversity changes have not been consistent. Suskun et al. observed significant differences in both Shannon diversity and community composition among children with PIMS, acute COVID-19, and healthy controls (42). Romani et al., in a cohort containing only four PIMS cases, identified a distinct microbial configuration characterized by enrichment of several inflammation-associated genera (43). More recently, Franchitti et al. reported compositional differences between children with severe COVID-19 and PIMS using 16S rRNA sequencing and targeted molecular analyses (23). Therefore, individual microbial taxa should not yet be regarded as universal biomarkers of PIMS. Taken together, these findings suggest that taxonomic reorganization may be a more reproducible feature than a uniform reduction in microbial richness across pediatric SARS-CoV-2-related inflammatory conditions. Looking specifically for taxonomic members, we found the abundance of *Prevotella copri* clade C*, Duodenibacillus massiliensis, Phascolarctobacterium succinatutens, and Enterocloster bolteae* differed markedly between patients with PIMS and controls, with all taxa showing increased abundance in the PIMS group. The common Prevotella genus inhabits the gut and is beneficial in terms of degradation of complex polysaccharides and its participation in the gut-immune system. However, overgrowth of certain Prevotella species has been associated with inflammatory processes and localized infections. Other taxa showing increased abundance in the PIMS group were taxonomically characterized only within the last decade; consequently, the available literature remains insufficient to establish a clear association between these species and the pathology under investigation. *Duodenibacillus massiliensis* (44) belongs to the family *Sutterellaceae* (first described in 2010), whose principal genus is *Sutterella*. Members of this family are strictly anaerobic microorganisms that inhabit the human gut. However, related species have also been isolated from clinical specimens, including abdominal abscesses, blood cultures, and other infected tissues, suggesting a potential role as opportunistic pathogens. By the way, *Enterocloster bolteae* belongs to a genus of anaerobic, spore-forming, spindle-shaped bacilli that utilize simple carbohydrates for growth and commonly inhabit the human gut. Similar to other members of the genus, it has been detected in diseases fluids and tissues, supporting its characterization as potential opportunistic pathogen under certain conditions (45). Along with *P. copri, D. massiliensis,* and *E. bolteae* described in this work, microbes of genera *Streptococcus, Rothia, Veillonella, and Actinomyces* can be also highlighted as opportunistic pathogens with a marked presence in patients with PIMS (20). Romani et al. described enrichment of *Veillonella*, *Clostridium*, *Dialister*, *Ruminococcus*, and *Streptococcus* in the small PIMS subgroup, whereas Suskun et al. reported broader differences involving members of the *Bacteroidetes* and *Firmicutes* phyla (42, 43). The absence of complete taxonomic concordance across studies may reflect differences in age, geography, diet, SARS-CoV-2 variant, disease severity, timing of stool collection, previous treatment, and sequencing methodology.

Following taxonomic profiling of the microbial communities, enzyme-coding genes were analyzed as part of the metagenomic workflow. Whereas earlier PIMS investigations relied predominantly on 16S rRNA sequencing and therefore provided taxonomic information or indirectly inferred functional profiles. More recent studies have also reported altered predicted immune-related pathways and fecal metabolomic profiles in pediatric COVID-19, but not specifically in PIMS. Our study therefore adds a distinct layer of evidence by identifying metagenomically encoded enzymes, functional annotations, and metabolic pathways associated with PIMS. The analysis specifically targeted enzymes directly or indirectly associated with PIMS, as well as those involved in key metabolic pathways implicated in the development and regulation of inflammatory responses. The mRNA encoding aconitate hydratase (EC: 4.2.1.3) was significantly more abundant in the PIMS group than in the control one (Figure 4). Aconitate hydratase is involved in the synthesis of itaconitate, which occurs in response to inflammatory stimuli, such as lipopolysaccharides (46). In contrast, protein-tyrosine phosphatase (PTP) (EC: 3.1.3.48) reported higher values in the control group than in children with PIMS (Figure 6). This enzyme is related to the inflammatory process, as it plays a critical role in coordinating the signaling networks that maintain lymphocyte homeostasis and direct activation. PTPs are also involved in immune cell signaling, including the phosphorylation and activation of STAT proteins, which are effectors of cytokine signaling. It also has a role in protecting the function of the intestinal epithelial barrier and regulating innate and adaptive immune responses and gut homeostasis. PTP dysfunction results in aberrant and uncontrolled immune responses, leading to chronic inflammatory conditions (47). These functional results extend previous pediatric microbiome studies by examining the microbial gene repertoire directly through shotgun metagenomics.

An additional limitation of this study is that most patients had received antibiotic therapy before fecal sample collection, reflecting routine clinical management during the evaluation of suspected PIMS. In addition, obesity was present in a proportion of the cohort. Both antibiotic exposure and obesity are well-recognized modulators of gut microbial composition and function and therefore may have contributed to the taxonomic and functional differences observed independently of PIMS itself. Given the limited sample size, adjustment for these potential confounders was not statistically feasible without substantially reducing analytical robustness. Consequently, the microbial signatures identified here should be interpreted as reflecting the overall gut microbiome profile observed in children with PIMS under real-world clinical conditions rather than changes attributable exclusively to the syndrome.

Despite these limitations, comparison with the available pediatric literature indicates that gut microbial alterations are reproducibly associated with SARS-CoV-2-related conditions, although the specific taxonomic signatures remain heterogeneous across studies. The present work advances this evidence by showing that children with PIMS also exhibit differences in the encoded microbial functional repertoire, including enzyme-coding genes and metabolic pathways not previously reported in this context. Nevertheless, these findings should be regarded as exploratory associations, and the identified taxa and functional features should be considered candidate biomarkers rather than definitive disease signatures. Larger multicenter studies incorporating longitudinal sampling, detailed dietary and medication data, complementary metatranscriptomic, metaproteomic and metabolomic measurements, together with appropriately powered statistical validation, will be required to determine whether these functional features precede PIMS, arise as a consequence of systemic inflammation or treatment, or persist after clinical recovery.

## 5 Conclusion

This exploratory study represents one of the first collaborative efforts in Latin America to characterize the gut microbiota and putative functional landscape in a cohort of children with PIMS associated with SARS-CoV-2 infection using a comprehensive metagenomic approach. Despite the limited sample size (a limitation inherent to the low prevalence of this condition), the results obtained provide preliminary but significant evidence of microbial unbalance and putative functional alterations associated with PIMS compared with clinically healthy controls. While alpha diversity did not differ significantly, microbial community composition and several metagenomically inferred functional features—including enzyme-coding genes and metabolic pathways—showed significant differences between groups, suggesting that inferred alterations in the microbial functional repertoire are associated with this syndrome.

These findings extend previous taxonomic observations by providing an integrated characterization of microbial composition and inferred functional potential in pediatric PIMS.

Altogether, this study provides a model for other investigations related to post infectious inflammatory disorders and inflammatory diseases aimed to explore mechanistic observations. Given the exploratory, cross-sectional design and limited sample size, these results should be considered hypothesis-generating and require validation in larger, longitudinal, multicenter cohorts incorporating complementary approaches such as metatranscriptomics, metaproteomics, metabolomics, and host immune profiling to determine their biological and clinical relevance.

## Data Availability

The raw sequencing data generated in this study are publicly available in the National Center for Biotechnology Information Sequence Read Archive (SRA) under BioProject accession PRJNA1455169.
